# Modelling the disappearance of coarse woody debris, following a land clearing event

**DOI:** 10.1186/s13021-021-00199-y

**Published:** 2021-12-07

**Authors:** Matthew J. Pringle, Steven G. Bray, John O. Carter

**Affiliations:** 1 Department of Environment and Science, GPO Box 2454, Brisbane, QLD 4001 Australia; 2 Department of Agriculture and Fisheries, GPO Box 267, Brisbane, QLD 4001 Australia

**Keywords:** Deforestation, Decay, Agriculture, Logit, Spline, Uncertainty

## Abstract

**Background:**

Land clearing generates coarse woody debris (CWD), much of which ultimately becomes atmospheric CO_2_. Schemes for greenhouse gas accounting must consider the contribution from land clearing, but the timing of the contribution will have large uncertainty, due to a paucity of knowledge about the rate of CWD disappearance. To better understand above-ground CWD disappearance following a land clearing event—through the actions of microorganisms, invertebrates, wildfire, or deliberate burning—we combined statistical modelling with an archive of semi-quantitative observations (units of CWD %), made within Queensland, Australia.

**Results:**

Using a generalised additive mixed-effects model (median absolute error = 14.7%), we found that CWD disappearance was strongly influenced by the: (i) number of years elapsed since clearing; (ii) clearing method; (iii) bioregion (effectively a climate-by-tree species interaction); and (iv) the number of times burned. Years-since-clearing had a strongly non-linear effect on the rate of CWD disappearance. The data suggested that disappearance was reverse-sigmoidal, with little change in CWD apparent for the first three years after clearing. In typical conditions for Queensland, the model predicted that it will take 38 years for 95% of CWD to disappear, following a land clearing event; however, accounting for uncertainty in the data and model, this value could be as few as 5 years, or > 100 years. In contrast, due to an assumption about the propensity of land managers to burn CWD, the official method used to assess Australia’s greenhouse gas emissions predicted that 95% of CWD will disappear in < 1 year.

**Conclusions:**

In Queensland, the CWD generated by land clearing typically takes 38 years to disappear. This ultimately implies that a key assumption of Australia’s official greenhouse gas reporting—i.e. that 98% of CWD is burned soon after a clearing event—does not adequately account for delayed CO_2_ emissions.

## Background

Coarse woody debris (CWD)—comprising standing dead trees, and the stems, branches, and stumps of fallen trees [1–3]—can be created naturally (e.g. tree death, canopy damage) or anthropogenically (e.g. pruning, harvesting, land clearing). CWD plays a role in various ecosystem functions, such as nutrient cycling [4–6]; regulation of soil moisture and temperature [7, 8]; and provision of microhabitats for small animals [9–11] and grazing-sensitive plants [12]. CWD also helps to maintain the natural functioning of streams [13], and adds to the fuel load for wildfire [14–16].

CWD disappears by a combination of microbial decomposition, consumption by invertebrates (particularly termites), fire, or physical degradation [3, 17, 18]. The ultimate product of much CWD disappearance is CO_2_ emission into the atmosphere, which makes the study of CWD interesting from the perspective of climate change, and greenhouse gas inventory. Many studies in different parts of the world have described the nature of CWD disappearance, and its drivers. Exponential decay is often used to model the disappearance of CWD with time, having been applied to study sites from the northern hemisphere [19–21], the southern hemisphere [22–24], and globally [25]. Mackensen et al. [17] found that temperature, rather than moisture, is the key influence on the rate of CWD disappearance, a finding that has been echoed by studies since [25–27]. CWD disappearance is also influenced by tree species [28, 29], wood diameter [22], fire [19, 30], and soil factors such as clay type [31].

Few studies have given attention to the CWD generated by land clearing events. Mackensen and Bauhus [32] synthesised the knowledge used to assess the contribution of land clearing to greenhouse gas emissions in Australia. Land clearing has been shown to create a large spike in CWD stocks [19, 33]. Navarrete et al. [33] showed that a relatively high grazing intensity is associated with relatively fast CWD disappearance, due to movement of CWD by heavy machinery in more-productive grazing lands. In the context of land clearing, we expand the conventional definition of CWD to include any coarse roots that are (perhaps only partially) extracted from the ground during the clearing event.

In Australia, the national greenhouse gas inventory (NGGI) uses a spatially applied process model known as FullCAM (‘full carbon accounting model’), to consider the role of land clearing and CWD [34, 35]. The NGGI combines: (i) remote sensing-based estimates of the spatial extent and timing of land clearing events; (ii) estimates of pre-clearing forest biomass (based on climate, management and other site attributes); and, (iii) assumptions on the immediate and lagged disappearance of the CWD and soil carbon pools during the next 50 years. At the time of clearing, ‘live’ biomass estimated for the major vegetation groups within a mature forest becomes ‘dead’ biomass, and is split into components of stem, branch, bark, leaf, coarse-root and fine-root. The NGGI then calculates the disappearance of each component individually, with different decay rates applied to each, depending on whether the component enters the ‘standing dead’ or ‘debris’ pools [36]. On clearing, the stem, branch, bark, and leaf components each enter the standing-dead pool, which decomposes relatively slowly, due to an assumed lack of contact with soil-borne decomposers. Each month after clearing, a small proportion of the standing-dead pool enters the debris pool, which is assumed to be in better contact with the soil and hence decomposes relatively fast. The coarse- and fine-root components, due to an assumed proximity to the soil, decompose solely from within the debris pool. For the NGGI’s reporting class ‘Land converted to grassland’—the class most relevant to this study—the following management practices are assumed [35]:

- The principal method of land clearing involves extraction of root material (i.e. tree-pulling).
- Following tree-pulling, and 6–10 months of curing, CWD is pushed into piles for burning. The standing dead pool is assumed to have a 98% combustion efficiency, but in the debris pool combustion efficiencies are 90% for stems and branches, 95% for bark and leaf, 80% for coarse roots, and 70% for fine roots.
- All remaining CWD decomposes naturally.

Under this reporting class the state of Queensland, Australia, emitted 16,504 Gg CO_2_-e in 2019 [37]. This was 3% of national emissions, down from 14% in 1990. The assumption that 98% of standing dead CWD is burned is, in our experience, too large a value for Queensland’s landscape. The effect will be to overestimate the rate of CWD disappearance, and subsequently cause a short-term overestimation of the amount of greenhouse gas emissions attributable to land clearing, and an underestimation of emissions in the decades that follow. In this paper, we evaluate the assumptions of the NGGI in regard to land clearing, by using a previously unpublished archive of semi-quantitative field observations of CWD disappearance.

### Field assessment of CWD disappearance

Between 1988, when records started, and 2018, 24-million ha of woodland and forest have been cleared or re-cleared in Queensland, 93% of which was for agricultural purposes [38]. Land clearing for agriculture in Queensland commonly occurs by tree-pulling (done with the aid of heavy machinery), or by application of arboricide. Subsequent management may involve: doing nothing and allowing the CWD to naturally decompose; or pushing the CWD into a pile that can either be retained or burned. There are less invasive, more selective land clearing options, e.g. fodder harvesting or forestry practice, but for the purpose of this study we consider only broadscale clearing.

Queensland’s Statewide Landcover and Trees Study (SLATS) supports legislation that regulates land clearing. SLATS uses automated and manual methods to regularly map the extent of woody vegetation that has been cleared [39]. A key component of the SLATS method for a number of years was field verification, to clarify areas of uncertainty in the remotely sensed mapping. Verification sites were generally chosen for their easy roadside access. When visiting a verification site, trained operators were asked to provide a visual, semi-quantitative estimate of the percentage of CWD that had disappeared since the clearing event, assessed with the aid of Table 1, to minimise inter-operator error.

**Table 1:** Classes used for visual estimation of the disappearance of coarse woody debris (CWD)

Disappearance (% of original CWD)	Description
0	No timber or branches or leaves gone
5	Only leaves gone
10	Branches < 2-cm diameter and bark gone
20	Branches < 10-cm diameter gone
30	All small branches gone
40	All small branches and some large branches gone
50	All small branches and pre-clearing trunks or large branches gone
60	Pre-clearing trunks one-quarter gone
70	Pre-clearing trunks one-half gone
80	Pre-clearing trunks three-quarters gone
90	Pre-clearing trunks almost all gone
95	All cleared for pasture, roadside verge, or houseblock
100	All cleared for crop or buildings

The aim of this study was to use the field observations of SLATS to train a statistical model that relates the disappearance of CWD to various explanatory variables. Such a model will help to evaluate the current understanding about how CWD contributes to Australia’s national greenhouse gas inventory, but will also help to improve understanding of nutrient cycling, ecosystem biodiversity, and soil function.

## Methods

### Pre-processing

**Fig. 1:**
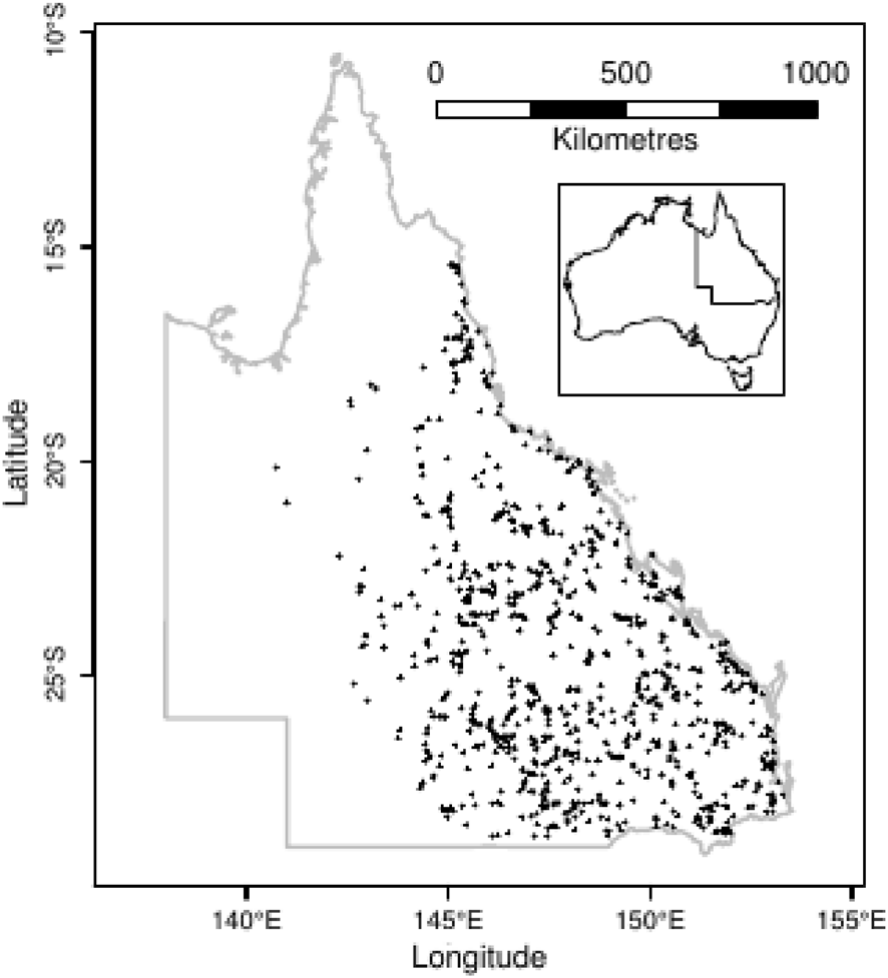
Locations of 1109 baseline sites in the state of Queensland, Australia, where in the years following a land clearing event, at least two observations were made of the disappearance of coarse woody debris. Inset: the location of Queensland within Australia

Within the digital archive of SLATS there were 8400 observations of CWD disappearance, collected since 1999. After stringently filtering for omissions and errors in the metadata associated with each observation we reduced that number to 3047. Omissions and errors came in various forms, e.g. inconsistent formatting, typographical errors, unreliable space–time locations, duplicated records, absence of an accompanying photo, or an excessive mismatch in the timing of an observation with the timing of its photo. Of the 3047 observations of CWD disappearance, many were revisits to a baseline site. Observations were grouped by the *n* = 1109 baseline sites (Fig. 1); i.e. each baseline site was revisited an average of 2.7 further times. Those parts of Queensland with no baseline sites were either too remote, or had experienced little land clearing.

**Fig. 2:**
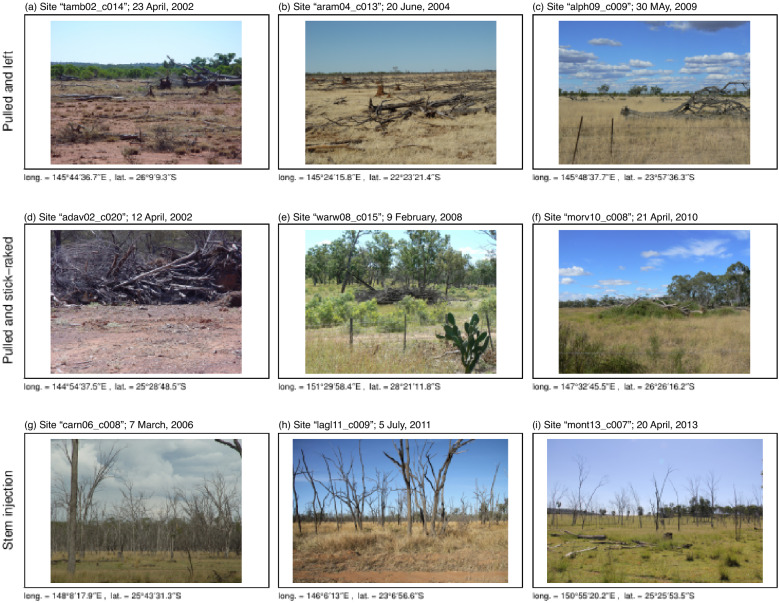
Exemplar photographs of the main types of land clearing in Queensland

Relevant components of the metadata for this study were the clearing method, and the SLATS reporting period. Trained operators determined the clearing method during field verification. Using expert knowledge, we aggregated the various clearing methods by the hypothesised rate of CWD disappearance associated with each (denoted by variable *c*, Table 2). The majority of the *n* baseline sites were associated with the clearing method ‘Pulled and left’, i.e. trees are up-rooted and the CWD naturally decays where it lies (Fig. 2a–c). We hypothesised this class has an intermediate rate of CWD disappearance, and combined these observations with the < 1% of observations that had an unidentifiable clearing method. The next-most-populous clearing method was ‘Pulled and stick-raked’, i.e. trees are up-rooted then moved into piles, possibly for burning (Fig. 2d–f). We hypothesised this clearing method has a relatively fast rate of disappearance. The hypothesised ‘slow’ methods of CWD disappearance were infrequently observed (4% of observations), but most often associated with stem-injection of arboricide (Fig. 2g–i). Note that the clearing method at baseline was sometimes adjusted by revisiting SLATS operators. For example, ‘Pulled and left’ observed at baseline could eventually change to ‘Pulled and stick-raked’ upon revisit. In this case, we set the latter as the clearing method.

**Table 2:** The hypothesised rate of disappearance of coarse woody debris, *c*; *n* is the number of sites

*c*	Proportion of *n*	Subsumed clearing methods (decreasing prevalence)
Intermediate	0.60	Pulled and left; unidentified
Fast	0.36	Pulled and stick-raked; blade-ploughed; selectively logged; thinned; grazing fodder
Slow	0.04	Stem-injection of arboricide; natural death; herbicide spray-drift

The SLATS reporting period defines the window of time, between a pair of satellite-image acquisition dates, when the land clearing event likely occurred. Currently, a reporting period is approximately one year, but depends on cloud cover and the satellite imagery available. Before 1999, reporting periods were, at best, two years, due to the resources then available. For the purpose of this study it was necessary to, where possible, narrow the clearing window associated with each of the *n* baseline sites. We did this according to the procedure described in Appendix.

### Statistical modelling

In broad terms, our modelling framework considered: (i) longitudinal effects in the CWD observations; (ii) errors inherited from the explanatory variables; and (iii) how predictions had to be within the interval (0%, 100%).

We used a generalised additive mixed-effects model (GAMM) to describe CWD disappearance as a function of various explanatory variables (fixed effects), and the *n* sites (random effects), with $j = 1, \ldots ,m_{i}$ observations at the *i*th site. The form of the GAMM was:

1 $${\text{logit}}\left( {y_{i,j} } \right) = \beta_{0} + \left\{ {\mathop \sum \limits_{k = 1}^{p} f_{k} \left( {X_{i,j,k} } \right)} \right\} + q_{i} + \varepsilon_{i,j} ,$$

where: $y_{i,j}$ was the *j*th observation of CWD disappearance in the *i*th site (converted to ‘% remaining’, i.e. the complement of disappearance); $\beta_{0}$ was the model’s intercept parameter; $X_{i,j,k}$ was the value of the explanatory variable *k* that was associated with $y_{i,j}$; $f_{k}$ was a smooth function of explanatory variable *k*; $q_{i}$ was the value of the random effect for the *i*th site, distributed as ${\mathcal{N}}\sim \left( {0, \varphi^{2} } \right)$; and $\varepsilon_{i,j}$ was the model’s error, distributed as ${\mathcal{N}}\sim \left( {0, \sigma^{2} } \right)$. The random-effect term is a way of controlling for different sources of variation, and implies that we expect observations from within the same site to be more closely related than observations from different sites. Without the random-effect term, the model reduces to a conventional generalised additive model, and $\varphi^{2}$ is subsumed by $\sigma^{2}$. Note the logit transformation applied to $y_{i,j}$, which was done to help $\varepsilon_{i,j}$ behave like a draw from a normal distribution. To enable the logit transformation—and to help emphasise the overall trend—when CWD disappearance in the *i*^th^ site was observed at 100% (i.e. completely gone), it was replaced with 99% for that year, and then imputed at 99% at 27 years after clearing (the largest value in the dataset). The GAMM was fitted with the gamm function of the mgcv library [40] of the R statistical software [41]. Residual maximum likelihood [42] was used to fit the model parameters.

Explanatory variable *c* was introduced above, but various others were investigated for their possible relation with CWD disappearance (Table 3). Explanatory variable *t* was the decimal years since clearing, while *r*, *v*, *g*, and *a* integrated various aspects of climate, fire, and soil variability. We calculated explanatory variables *r*, *v*, and *g*—respectively the proportion of rain days, the mean daily vapour pressure deficit, and the number of times burned—based on the number of days between when the clearing event occurred (see below) and when the site was visited by SLATS operators. The number of times burned was found by interrogating an archive of historical fire scars, detected in Landsat satellite imagery [43]. Like *c*, *b* was a categorical explanatory variable, but was derived from an Australia-wide surface of delineated bioregions [44]. Preliminary modelling (not shown) suggested that certain bioregions were sampled too rarely to have an effect; these were recoded to a member of *b* that was neighbouring or more densely sampled (Table 4).

**Table 3:** Explanatory variables for the generalised additive mixed-effects model; ‘s. c.’ is ‘since clearing’, and ‘NA’ indicates that the variable is categorical

Name	Description	Unit	Source
*c*	(See Table 2)	NA	Field operators
*b*	(See Table 4)	NA	[44]
*t*	Decimal years s. c	yr	Field operators
*r*	Proportion of rain days s. c	(unitless)	[46]
*v*	Mean daily vapour pressure deficit s. c	hPa	[46]
*g*	Number of times burned s. c	(unitless)	[43]
*a*	Clay content of soil surface (0–5 cm)	%	[60]

**Table 4:** Aggregated bioregions, *b*, used for modelling; *n* is the number of sites, and ‘NA’ means that no other bioregion was subsumed into *b*

*b*	Proportion of *n*	Additional subsumed bioregion(s)	Mean daily temperature, 1986–2014 (°C)	Median annual rain, 1986–2014 (mm)
Brigalow Belt North	0.19	Central Mackay Coast	25.1	608
Brigalow Belt South	0.43	Darling Riverine Plains; Desert Uplands, Einasleigh Uplands; Nandewar; New England Tablelands	24.6	584
Cape York Peninsula	0.02	Gulf Plains	26.5	1330
Mitchell Grass Downs	0.06	NA	25.7	334
Mulga Lands	0.19	Channel Country	25.1	271
South East Queensland	0.09	NA	24.0	896
Wet Tropics	0.02	NA	25.1	1923

Climate statistics were calculated from the daily Queensland-wide surfaces held in the SILO database [46]

From Table 3, it was necessary to identify the best combination to serve as *X* in Eq. (1). Rather than naively test all 127 possible combinations, we limited ourselves to 37 combinations, settled on by a subjective, backwards step-wise method: the most complicated model (‘Model 1’) was fitted first, and the results used to guide which explanatory variable(s) to drop for Model 2, and so on. We considered the presence of *t* to be mandatory throughout, except in Model 37, which was a control with no explanatory variables. Table 5 presents the most notable combinations tested. If *c* or *b* appeared in the GAMM, their most populous class (*c* = ‘Intermediate’; *b* = ‘Brigalow Belt South’) was incorporated into parameter $\beta_{0}$. For each continuous explanatory variable in Table 5, $f\left( . \right)$. of Eq. (1) took the form of a penalised cubic regression spline [40], which we represent herein as ‘$\mathrm{s}\left(.\right)$’. The spline associated with *t* could be split by the classes of *c*, i.e. the shape of the spline over time was allowed to vary by clearing method. When the split is present, this spline is denoted $\mathrm{s}\left(t|c\right)$; without the split it is $\mathrm{s}\left(t\right)$. For $\mathrm{s}\left(t\right)$ and $\mathrm{s}\left(t|c\right)$, we used the default basis dimension of 10. For ${\text{s}}\left( a \right)$, ${\text{s}}\left( r \right)$ and ${\text{s}}\left( v \right)$, preliminary analysis (not shown) suggested to alter the basis dimensions to 6, 15, and 15, respectively, where the smaller the value, the more stiff the spline. We expected *r* and *v* to have an interactive effect on ${\text{logit}}\left( y \right)$, so when these explanatory variables appeared in the GAMM together, we replaced them with ${\text{s}}\left( {r,v} \right)$, a tensor product-based smooth of a penalised cubic regression spline that considered the variables jointly. The dimension of this joint spline was six per variable. We regarded *g* as a strictly linear term, because it had only three unique values in the dataset (i.e. 0, 1, or 2). Note that Model 37, because it had no explanatory variables to smooth, was fitted with the lmer function of R’s lme4 library.

**Table 5:** Some of the combinations of explanatory variables tested for the generalised additive mixed-effects model in Eq. (1), and the performance when applied to withheld validation data

Model	Combination of explanatory variables	PV	CCC	PCP	MAE	${\varvec{L}}^{2}$
1	$c + b + {\text{s}}\left( {t|c} \right) + {\text{s}}\left( {r,v} \right) + g + {\text{s}}\left( a \right)$	0.33	0.47	0.80	13.7	17.6
2	$c + b + {\text{s}}\left( {t|c} \right) + {\text{s}}\left( {r,v} \right) + g$	0.33	0.48	0.81	15.6	14.7
**7**	${\varvec{c}} + {\varvec{b}} + {\mathbf{s}}\left( {{\varvec{t}}|{\varvec{c}}} \right) + {\varvec{g}}$	**0.32**	**0.46**	**0.82**	**14.7**	**10.5**
12	$c + b + {\text{s}}\left( {t|c} \right)$	0.32	0.45	0.81	14.0	14.2
13	$c + {\text{s}}\left( {t|c} \right) + {\text{s}}\left( {r,v} \right)$	0.31	0.45	0.83	14.9	11.5
24	$b + {\text{s}}\left( t \right) + {\text{s}}\left( {r,v} \right)$	0.30	0.44	0.82	16.6	18.8
36	${\text{s}}\left( t \right)$	0.25	0.36	0.83	16.0	27.0
37	(none)	0.00	0.00	0.91	25.8	33.9

Function ‘${\text{s}}\left( . \right)$’ indicates a penalised cubic regression spline. Key to the explanatory variables: *c* = clearing method; *b* = bioregion; *t* = decimal years since clearing; *r* = proportion of rain days since clearing; *v* = mean daily vapour pressure deficit since clearing; *g* = number of times burned since clearing; *a* = clay content of soil surface (0–5 cm). Refer to Tables 2, 3, 4 for more information on the explanatory variables. Key to the columns: *PV* proportion of variance explained (logit scale), *CCC*  concordance correlation coefficient (logit scale), *PCP* proportion of sites correctly predicted, *MAE*  median absolute error (original scale of %); $L^{2}$ Euclidean norm. Bold-face indicates the best overall model

We split the dataset so that 90% of sites were available for training a model, and the remaining sites were used for validation. The workflow applied to each of the 37 models (Box 1) accounted for two major sources of uncertainty: one relating to the explanatory variables, and the other relating to model parameters. In regard to explanatory variables, if the years-since-clearing could only be determined to within, say, a 6-month window, then *t*, *r*, *v*, and *g* could all vary more compared with a site where years-since-clearing was accurate to within a one-month window. To address this issue, we calculated a lower and upper bound for each value of these explanatory variables, based on the earliest and latest dates for years-since-clearing (Appendix). These bounds served as parameters from which we drew a set of uniform random deviates (Box 1, step 2), which were then used as the explanatory variables for the model. In the case of *g*, only the integer part of each univariate deviate was considered. Explanatory variable *a*, i.e. clay content of the top 5 cm of soil, was also used in a manner that accounted for its uncertainty, except that the predicted clay content and its associated 95% prediction interval were used to define a triangular, not uniform, distribution. Each model was fitted 100 times with randomly perturbed explanatory variables. In regard to model parameters, we used numerical simulation to combine the different sources of variation: i.e. step 7b of Box 1 accounted for uncertainty due to the model’s fixed effects; step 7c accounted for uncertainty due to a validation site; and 7d accounted for residual uncertainty. The sum of the three simulated components formed a distribution for the prediction (logit scale). In total, at the end of the procedure in Box 1, every validation datum was associated with 10,000 simulated predictions, for all 37 models.

Box 1: The workflow for modelling dataset ‘z’

- Training:
  1. Subset the training sites from ‘z’
  2. Perturb the explanatory variables in the training subset
  3. Fit the model using the perturbed explanatory variables
  4. Save the model
  5. Repeat steps 2–4 a further 99 times
- Validation:
  6. Subset the validation sites from ‘z’
  7. For the *i*th of the 100 models:
    - Perturb the explanatory variables in the validation subset
    - Draw 100 random deviates from the distribution ${\mathcal{N}}\left( {\hat{p}_{j} ,{\text{se}}\left[ {\hat{p}_{j} } \right]^{2} } \right)$, where $\hat{p}_{j}$ was the value predicted by the model for the *j*th validation datum, and ${\text{se}}\left[ {\hat{p}_{j} } \right]$ was the corresponding standard error
    - For the *j*th validation datum, draw 100 random deviates from the distribution ${\mathcal{N}}\left( {0,\varphi^{2} } \right)$ (Eq. (1))
    - For the *j*th validation datum, draw 100 random deviates from the distribution ${\mathcal{N}}\left( {0,\sigma^{2} } \right)$ (Eq. (1))
    - Store the simulated values as b + c + d
  8. Summarise the 10,000 simulated values for each validation datum

We used the median of the simulated predictions at each validation location to calculate, for each model, the proportion of variance explained by the predictions (PV) and the concordance correlation coefficient (CCC [45]) of predictions with observations (logit scale). The target value for each of these is 1.0. We also used the simulated predictions to calculate the proportion of validation sites correctly predicted (PCP), i.e. where all observed values for a site were inside a model’s 95% prediction interval (logit scale). The target value for PCP is 0.95, which indicates that the model adequately characterises the variability of the observed data. Finally, we calculated the median of the back-transformed simulated predictions at each validation datum, and used the resulting set of values to calculate the median absolute error (MAE) on the original scale of the data (i.e. %). The target value for MAE is zero.

To judge the overall best model we calculated the absolute difference of PV, CCC, PCP and MAE from their target values, then converted the outcomes to ranks. We then used the mean and standard deviation of the ranks for each model to calculate a Euclidean norm. The model with the smallest Euclidean norm was the best overall. This model we subsequently re-fitted with the entire complement of sitess, as per the description in Box 1.

### Alternative methods of calculating CWD disappearance

To further judge the performance of the GAMM, we applied two published models of CWD disappearance to the withheld validation sites: (i) the single-exponential decay function of [17], where the rate constant is itself a function of mean air temperature; and (ii) the NGGI method for characterising CWD disappearance. For (i), mean-air-temperature-since-clearing was randomly perturbed within a range of values, as for the explanatory variables described above. For (ii), we combined the FullCAM two-pool method [36] with the constants published in Table 6.52 (which partitions biomass in a tree at the time of clearing) and Tables 6.55a,b (which respectively decompose the ‘standing dead’ and ‘debris’ pools) of [35]. The NGGI regards biomass partitioning as a function of annual rain, so we first calculated a Queensland-wide surface of median annual rain (mm) between 1986 and 2014, from the SILO database [46]. We set the date of burning to 10 months after clearing, and continued monthly calculations until 100 years after the clearing event.

A lagged exponential function was proposed by [1] to describe CWD disappearance:

2 $$y_{t} = 100\left( {1 - \left( {1 - {\text{exp}}\left( { - \propto t} \right)} \right)^{N} } \right),$$

where: ${y}_{t}$ was CWD remaining (%) at *t* years since clearing; $\propto$ was the rate constant; and *N* controlled the time lag for disappearance (the larger the value, the larger the delay). We optimised $\propto$ and *N* to fit the median back-transformed predictions of the GAMM for certain exemplary land clearing scenarios in Queensland.

## Results

**Fig. 3:**
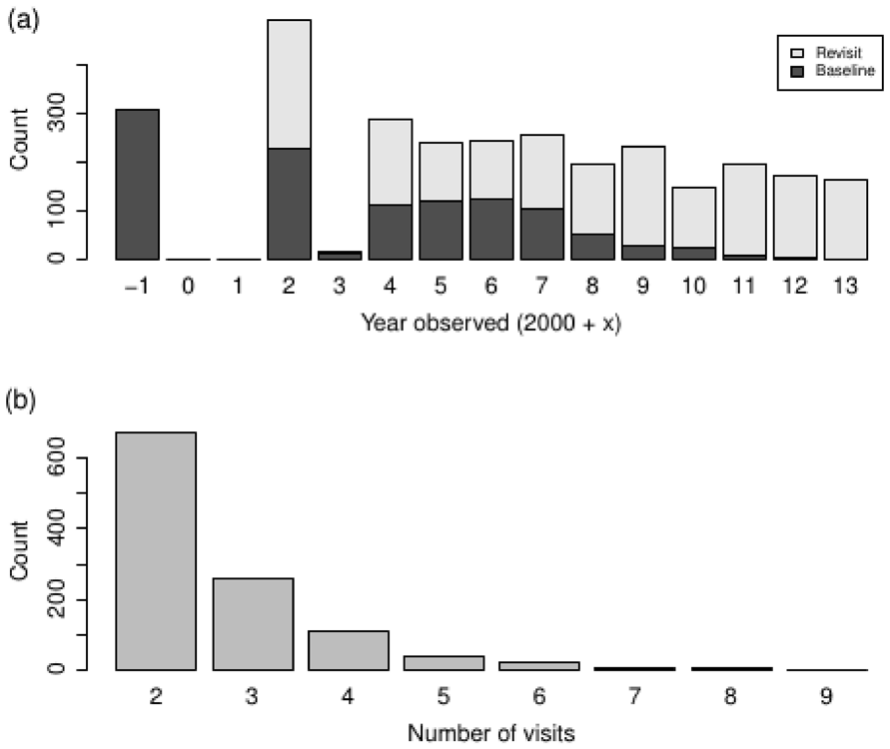
Histograms of the number of observations of the disappearance of coarse woody debris: **a** by year and the type of visit; and **b** by the number of visits per baseline site

Figure 3 presents histograms of different aspects of the SLATS observations of CWD disappearance. Over all sites (baseline and revisit), it is apparent that the intensity of the sampling (Fig. 3a) declined over time, and that no valid observations of CWD disappearance have been made since 2013. These reflect: (i) a long-term reduction in land clearing rates; and (ii) a contemporaneous shift in SLATS, towards increasingly confident use of the available satellite imagery. Eighty-four percent of sites were generally only revisited once or twice more, following the baseline visit (Fig. 3b). The average time between the baseline and the first revisit was 3.4 years.

**Fig. 4:**
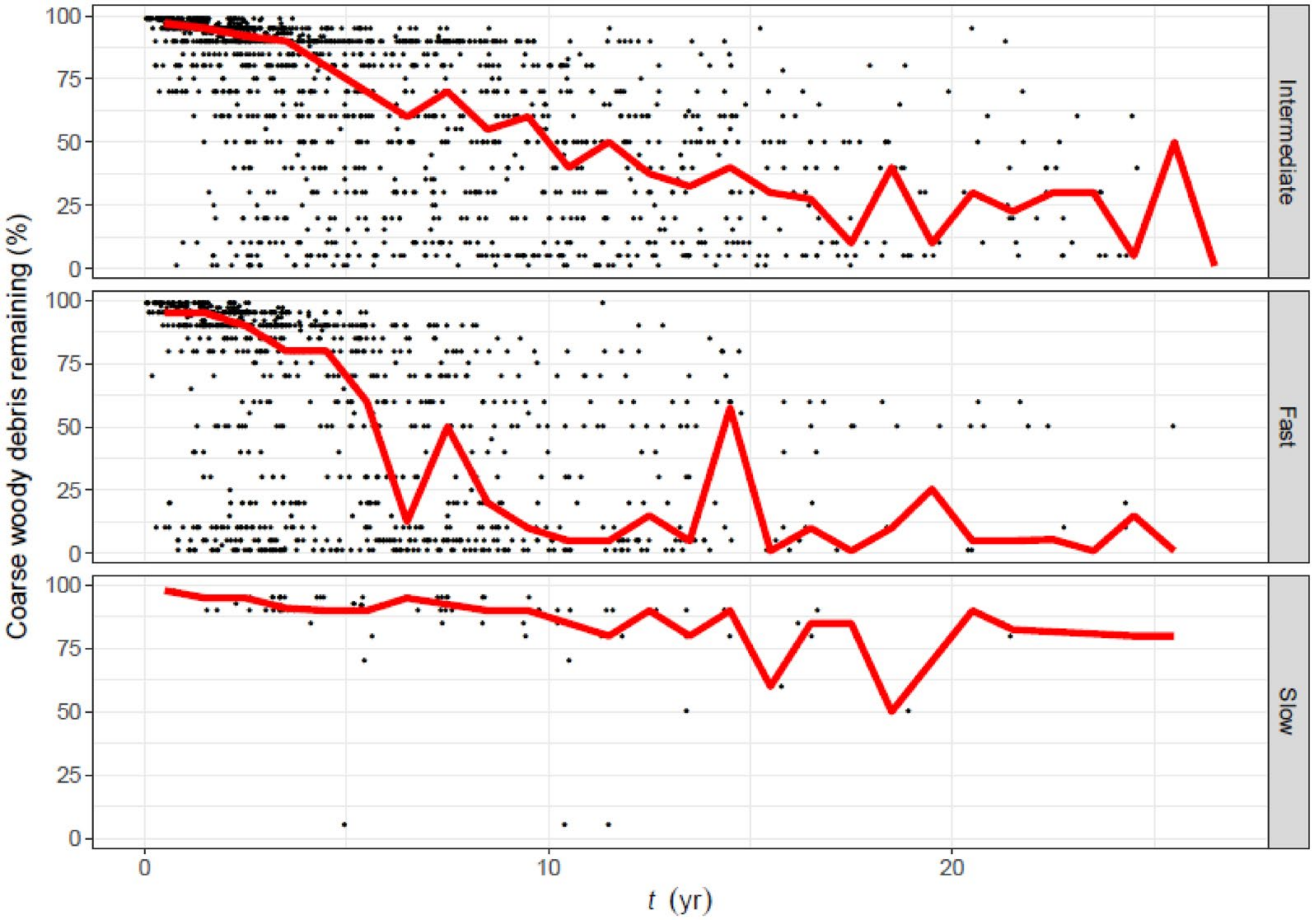
Observed disappearance of coarse woody debris, as a function of years since clearing (*t*), split by clearing method (Table 2). Clearing methods have been aggregated to reflect the hypothesised rate of CWD disappearance: Intermediate, Fast, or Slow (Table 2). To elucidate trends, the red line is a running median with a bin width of one year

We associate an intermediate rate-of-disappearance of CWD mainly with trees that have been pulled and left (Table 2). In this case, CWD tended to disappear slowly for the first three years, but then the rate increased such that, by 17 yr after clearing, much of the CWD was gone (Fig. 4). Relatively fast disappearance of CWD is mainly associated with trees that have been pulled and then stick-raked. In this case, CWD tended to disappear slowly for the first three years, but then the rate increased such that, by 10 yr after clearing, much of the CWD was gone. Relatively slow disappearance of CWD is mostly associated with stem-injection of aboricide. In this case, the CWD tended to decompose linearly, with relatively little change after 20 yr.

According to the withheld validation data, the best combination of explanatory variables was Model 7 (Table 5), which regards CWD disappearance as a function of years-since-clearing, clearing method, bioregion, and the number of times burned. Model 1 was better than Model 7 in regard to PV, CCC, and MAE, but these were countered by Model 7’s better performance with PCP. Model 13 was the second-best of the combinations tested, but replacing *g* with $\mathrm{s}\left(r,v\right)$ would not be wise in terms of parsimony; Model 12 would be a better second-choice combination, as it depends only on years-since-clearing and bioregion. In the absence of information about the clearing method, Model 24 was the best of those tested, but MAE increased from 14.7% to 16.6%. When years-since-clearing was the only explanatory variable (Model 36), PV and CCC decreased sharply. Relative to all other models, the poor performance of Model 37 suggests that the disappearance of CWD can be attributed to readily available environmental information. Model 37 recorded the largest PCP of 0.91. Bearing in mind the target PCP was 0.95, the consistent shortfall in Table 5 means that the models underestimate variance, and therefore prediction uncertainty; however, the values also partly reflect the conservative condition we applied, i.e. that all observations of a site had to be within the 95% prediction interval in order to contribute to the calculation of PCP.

When the single-exponential model of [17] and the NGGI method were applied to the validation sites, MAE was 20.2% and 58.6%, respectively. When the burning event of the NGGI method was omitted, MAE improved to 18.1%, but this was still not better than any of the GAMMs in Table 5.

**Fig. 5:**
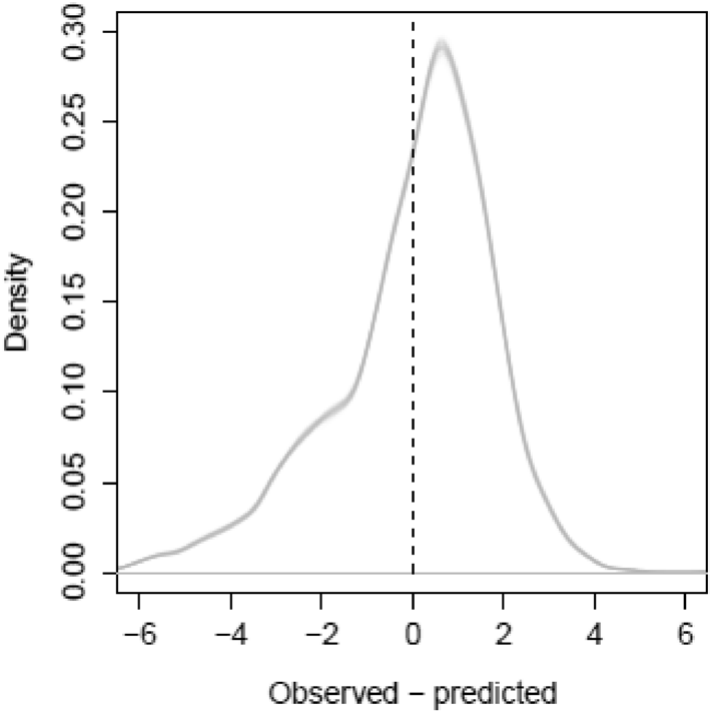
Probability densities for 100 sets of residuals of Model 7 (logit scale)

**Fig. 6:**
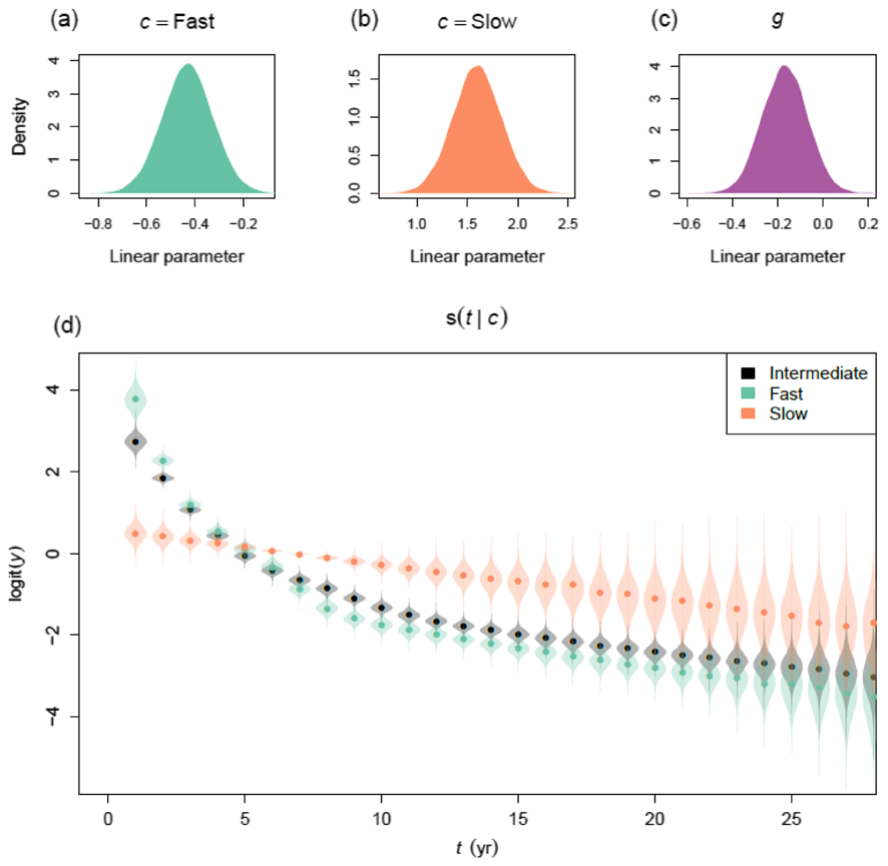
Aspects of the fixed effects of Model 7 (Table 5). Top row: probability densities of the linear parameters associated with *c* (method of clearing) and *g* (number of times burned). Bottom row: violin plot that shows the partial effect of the spline $\mathrm{s}\left(t|c\right)$ (i.e. years-since-clearing, conditional on clearing method)

For 100 fits of Model 7, the residuals were approximately normally distributed (Fig. 5). The slight negative skew suggests that either an important process is absent from the explanatory variables, or there was a bias in the operators’ interpretation of Table 1. Given the logit transform of *y*, the fitted linear parameters for *c* in Model 7 indicate the length of time it takes for 50% of CWD to decompose. Assuming the default clearing method is Intermediate (typified by ‘pulled and left’), CWD disappears to 50% significantly faster under the clearing methods typified by ‘Pulled and stick-raked’ (Fig. 6a), where the tails of the distribution suggest significance at $P<0.01$. For the same reason, under the clearing methods typified by stem injection of aboricide, CWD disappears significantly slower, significant also at $P<0.01$ (Fig. 6b). The predominantly negative sign of the linear parameter *g* was expected—the greater the incidence of fire, the faster the CWD disappears—but the effect was less strong than *c*, significant at $P<0.1$ (Fig. 6c). The partial effect of the spline $\mathrm{s}\left(t|c\right)$ was biologically sensible in that CWD disappeared with time (Fig. 6d). The disappearance was strongly non-linear, except when the land clearing method was ‘Slow’, which agreed with the exploratory plot in Fig. 4. Note that all probability densities in Fig. 6 were generated from 10,000 values, according to Box 1.

**Fig. 7:**
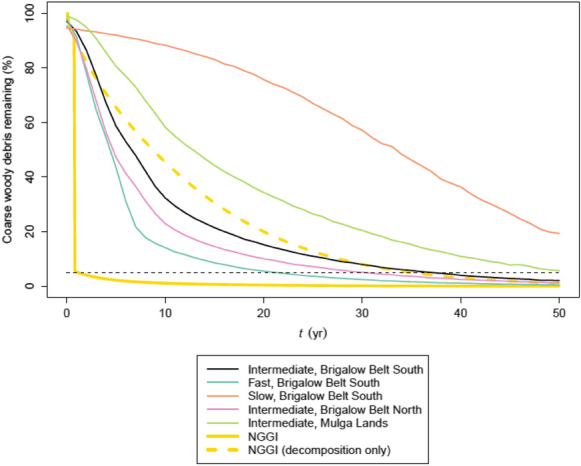
The disappearance of coarse woody debris, as a function of time, predicted by Model 7 (Table 5) for exemplar combinations of explanatory variables *c* (method of clearing) and *b* (bioregion). Note that the number of times burned was $g=1$ if *c* = Fast, but $g=0$ otherwise. For comparison, values calculated from Australia’s national greenhouse gas inventory (NGGI) are also shown in solid gold; the dashed gold line is the same, but with the assumptions about stick-raking and burning turned off. The dashed horizontal line is the threshold for 95% disappearance

Figure 7 illustrates how Model 7 predicts in various scenarios, when back-transformed from logit. For plotting clarity, we have omitted all 95% prediction intervals; however, the intervals are incorporated into Table 6, which presents, for the same scenarios, the median and the fewest years needed for 95% of CWD to disappear. Based on the SLATS data, a common scenario for land clearing in Queensland is that trees are pulled then left to decompose naturally, within the Brigalow Belt South bioregion (or one of its subsumed bioregions in Table 4). In this case, it will typically take 38 yr for 95% of CWD to disappear, with a lower bound of 5 yr. If trees of the Brigalow Belt South bioregion are pulled, stick-raked, then burned once, it will typically take 22 yr (lower bound = 4 yr). If tree stems in the Brigalow Belt South bioregion are injected with arboricide, they will typically take 68 yr for 95% of the CWD to disappear (lower bound = 24 yr). In the Brigalow Belt North bioregion, trees that are pulled and left will typically take 31 yr for 95% of CWD to disappear, with a lower bound of 4 yr. The principal climatic difference between the two bioregions is that Brigalow Belt North is 0.5 °C d^−1^ warmer than Brigalow Belt South (Table 4). In the Mulga Lands bioregion, trees that are pulled and left will typically take 53 yr for 95% of CWD to disappear (lower bound = 10 yr). The principal climatic difference between the bioregions is that Mulga Lands is 0.5 °C d^−1^ warmer and 313 mm of rain yr^−1^ drier than Brigalow Belt South (Table 4). In all scenarios, the upper bound for disappearance was > 100 yr. According to the NGGI it took < 1 yr for 95% of CWD to disappear. When the burning condition of the NGGI was omitted, the disappearance of CWD was much closer to the statistical models and the observations of Fig. 4, and corroborates the decrease in MAE noted above. In Table 6 we present the parameters of Eq. (2) that best fit the GAMM predictions of each scenario in Fig. 7, provided as a reference for future studies.

**Table 6:** The typical number of years for 95% of CWD to disappear, as predicted by Model 7 (Table 5), for exemplar combinations of clearing method (*c*), bioregion (*b*), and the number of times burned (g)

*c*	*b*	*g*	Number of years	$\propto$	*N*
Intermediate	Brigalow Belt South	0	38 (5)	0.091	0.891
Fast	Brigalow Belt South	1	22 (4)	0.213	1.325
Slow	Brigalow Belt South	0	68 (24)	0.057	3.828
Intermediate	Brigalow Belt North	0	31 (4)	0.117	0.846
Intermediate	Mulga Lands	0	53 (10)	0.060	1.170

The value in brackets is the fastest scenario for disappearance, given the model’s prediction uncertainty. Also shown are values for parameters $\propto$ and *N* of the lagged exponential function of [1] (Eq. (2)), which give the best approximation to the median predictions of Model 7

## Discussion

### On the CWD observations

We have used an archive of observations, accumulated over many years by SLATS, to explore the dynamics of CWD disappearance in Queensland, Australia. These data provide an efficient way to evaluate the current understanding of an important component of Australia’s greenhouse gas emissions: land clearing. Even though the data are semi-quantitative, and actual CWD biomass cannot be inferred, the pattern of disappearance provides a basis for comparison.

While the data are by no means an unbiased probabilistic sample, enough observations are available to suggest that, following a land clearing event, the majority of CWD in Queensland is left to decompose naturally (Table 2). When CWD is stick-raked the chance of it being ultimately burned increases, but we are unable to estimate anything more precise than an upper bound of 36% for the amount of CWD deliberately burned. In the set of observations that we excluded due to there being no revisit, the amount of stick-raking was 25%, which suggests that subsampling did not induce any serious fire-related bias into the analysis. If anything, SLATS’ roadside sampling over-represents stick-raking, because some land managers are motivated to present ‘clean’ land in areas of high public visibility. Ultimately, the upper bound of 36% deliberately burned is substantially smaller than the 98% assumed by the NGGI. In the proportionately large (80% by area [47]), less-fertile areas of Queensland, land is mainly cleared for grazing purposes. Land managers in these areas have little incentive to expedite the removal of CWD, because animals can still graze adequately, although mustering in the presence of much CWD is hazardous. The relatively fertile, though proportionately small, cropping and coastal areas of Queensland create an incentive for land managers to expedite the removal of CWD. Land intended for cropping is more likely to be stick-raked and burned, to enable cultivation and a reasonably prompt return on investment.

### Elaborating the different components of the model

We used a generalised additive mixed-effects model (GAMM) to predict logit-transformed CWD disappearance. It is worth elaborating the different components of this statement. ‘Mixed effects’ refers to how the model splits CWD disappearance into components associated with ‘fixed’ and ‘random’ effects. Fixed effects describe a deterministic response to an explanatory variable, while random effects describe a probabilistic response that (in this study anyway) we want to control for, but are not specifically interested in. In the context of this study, years-since-clearing is the key fixed effect, while we regard between-site differences as a random effect. A similar approach was taken by [20] to describe CWD disappearance, except that they considered between-species differences as a random effect. ‘Additive’ refers to how the different functions of the explanatory variables are added together to influence CWD. These functions are not necessarily linear, which we showed in Fig. 6d. ‘Generalised’ refers to how the errors of the model are assumed to follow a distribution that is not, as in other forms of regression, necessarily restricted to the normal distribution. We actually ignored this aspect of the GAMM, and applied a logit transform, which is commonly applied to models of proportions and percentages, to help the errors conform to a normal distribution (Fig. 5).

In regard to the logit transform, three advantages were apparent in this study. Firstly, it ensured that a back-transformed prediction would always fall within the interval (0%, 100%). Secondly, a back-transformed prediction at $t=0$ returned a biologically sensible value close to 100%. Thirdly, back-transformed predictions conformed with a visual trend in the observed data, i.e. CWD disappearance tended not to begin immediately, but rather had a delayed onset (Figs. 4, 7). We discuss this in more detail below.

### Interpretation of the results

We found that the best statistical model to explain the disappearance of CWD involved years-since-clearing, clearing method, bioregion, and the number of times burned. The years-since-clearing effect was strongly non-linear when the clearing method involved tree-pulling. The rate of disappearance increased when the tree-pulling was followed by stick-raking. Disappearance of poisoned trees was relatively slow. The number of years required for CWD to disappear (Table 6) is comparable with a study in the arid zone of South Australia [48]. Many studies have previously found that temperature and tree species are important factors that determine the variability in CWD disappearance [17, 25, 28], so it is not surprising that we found bioregion—an explanatory variable that effectively describes a climate-by-tree species interaction—to be important. In practical terms, the clearing method can be difficult to establish. ‘Pulled and left’ is the default clearing method of the model and, as discussed above, a reasonably safe assumption for much of Queensland. The model could be validly applied in other parts of Australia where the bioregion intersects Queensland. We do not recommend extrapolating the model elsewhere, on the basis that the already-large prediction uncertainty will only increase. Where the clearing method cannot be reliably established or assumed, we have shown that a reduced model comprising effects for only bioregion and years-since-clearing suffices (i.e. Model 12 of Table 5).

The model predicts relatively slow CWD disappearance in the first years after a land clearing event. This delayed onset was the principal reason that we eschewed the simple exponential decay models commonly used to describe CWD disappearance. Exponential decay, by definition, proceeds at a rate proportional to the available substrate, but implicitly assumes that the agent of decay—be it physical, chemical, or biological—can always act on the substrate, at any time. We contend that this is a strong assumption for the CWD generated by a land clearing event, and is not supported by our data (Fig. 4). The main tree species of the study region have relatively dense wood [49, 50], which, together with a predominantly semi-arid climate, slow the onset of decay by microorganisms and invertebrates. The delay may also be partly explained by changes to the species composition of termites that is caused by land clearing [51], or the time needed for CWD to dry. In some cases, the delay may simply be due to an absence of management intervention, particularly if, as noted above, the land is intended for grazing. Equation (2) was expounded by [1] to consider CWD disappearance through physical fragmentation (e.g. the action of gravity or insects), which, by its nature, takes time to manifest. In our study, we apply Eq. (2) more generally, with parameter $\propto$ integrating all forms of disappearance. The optimised parameter values in Table 6, though bound to the given scenarios, might conceivably act as a metamodel that informs a description of CWD disappearance simpler than the GAMM.

We have demonstrated that the rate of CWD disappearance is vastly different between our statistical model and the NGGI (Fig. 7). The NGGI method predicts almost-complete CWD disappearance in a single year following land clearing, due to assumptions around prompt stick-raking and burning. We have shown that Model 7 slightly over-predicts the disappearance of CWD in some circumstances (Fig. 5), but does reflect the broad trend (Fig. 4), and behaves similarly to a variant of the NGGI that considers decomposition only (Fig. 7) We suggest that those responsible for the NGGI now have, through our publicly available dataset, a further source of ground-based observations to help test assumptions. We further suggest the following research questions for future studies: (i) does the coarse-root component of CWD justify its presence in the debris pool, given that tree-pulling tends to partly extract it from the ground? (ii) do stick-raked piles of CWD decay faster because their microclimate encourages decomposition from within the pile, rather than just decomposing due to contact with soil? and (iii) can the burning of stick-raked CWD be detected in daily satellite imagery?

Our statistical model of CWD disappearance is potentially important from the perspective of greenhouse gas accounting, at a farm, regional—and even national—scale. The relatively slow predicted rates of CWD disappearance will impact the net greenhouse gas balance, and may also have an impact on the total greenhouse gas emissions that can be offset, particularly if a business—or Queensland’s grazing industry as a whole—tries to claim carbon neutrality. We acknowledge that, following a land clearing event, the majority of the carbon held in CWD will eventually be lost to the atmosphere, regardless of the rate of disappearance. However, when disappearance rates are relatively slow, losses can be more effectively offset by the growth and regrowth of woody vegetation [52], thus leading to larger carbon stocks on agricultural land than would otherwise be predicted. We must also acknowledge that land clearing contributes to non-CO_2_ greenhouse gases. Burning of CWD will generate—like wildfire—methane, carbon monoxide, and nitrous oxide [53], and termites generate methane [54]. These topics were beyond the scope of our study.

## Conclusions

We combined statistical modelling with an archive of semi-quantitative data, to study the disappearance of CWD (%) in Queensland, Australia, following a land clearing event. The median absolute error of the model was 14.7%. Disappearance was strongly influenced by years-since-clearing, the clearing method, bioregion, and the number of times burned. Years-since-clearing had a strongly non-linear effect on the rate of disappearance. Contrary to many other studies, our data did not support modelling by simple exponential decay; instead, disappearance was reverse-sigmoidal, with little change apparent for three years following a land clearing event. In typical conditions for Queensland—i.e. trees are pulled-and-left, the bioregion is (or is alike to) Brigalow Belt South, and the CWD remains unburned—we found that, following a land clearing event, it will take 38 yr for 95% of CWD to disappear. In contrast, due to an assumption about the propensity of land-managers to burn CWD, the official method used to report Australia’s greenhouse gas emissions predicted that 95% of CWD would disappear in < 1 yr. This result ultimately implies that official reporting incorrectly apportions the annual contribution of land clearing to Queensland’s CO_2_ emissions; presumably the same assumptions are applied elsewhere in Australia where environmental conditions are similar. Our statistical model suggests that the profile of land clearing emissions over time is much smoother than otherwise thought, which has implications for assessing change relative to emission baselines. We showed that CWD disappearance increased if CWD was formed into piles (possibly for burning), or if temperature increased and rainfall stayed constant. The rate of CWD disappearance slowed if the clearing method was stem-injection of arboricide, or if the bioregion was relatively dry.

## Figures and Tables

**Fig. 8:**
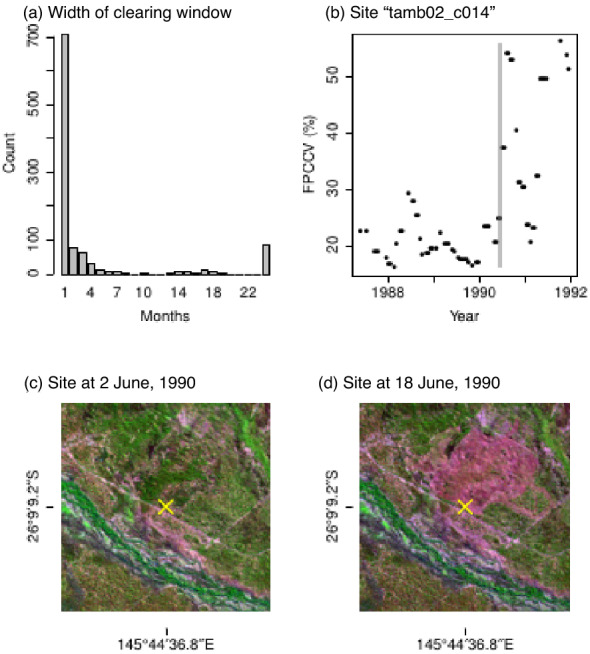
**a** Histogram of width-of-clearing-window, for 1109 baseline sites. **b** Time-series of the coefficient-of-variation of Landsat foliage projective cover (FPCCV), for an exemplar baseline site. The grey box is the site’s estimated clearing window, the single month of June, 1990. This clearing window is verified by the difference between panels **c** and **d**, which contain Landsat-5 TM images of surface reflectance (R = Band 5, G = Band 4, Blue = Band 3). The extent of the Landsat images is 5 km × 5 km, and the yellow cross in each is the spatial coordinate of the baseline site

## Data Availability

The pre-processed data and scripts to replicate the modelling are available at https://doi.org/10.6084/m9.figshare.14544645.v2.

## Appendix

We used the concept of image texture [55] to help narrow the clearing window, on the assumption that a land clearing event will, in general, disturb the space–time stability of the local environment.

For each baseline site we assembled a stack of intersecting Landsat images (30-m spatial resolution; 16-day temporal resolution) that spanned the SLATS reporting period. Spatially, the stack was restricted to a five-by-five block of pixels centred on the spatial coordinate of the baseline site. We then applied a statistical model pixel-wise to the stack, to estimate, for a unique observation date, foliage projective cover (FPC [56]) from the sextuple vector of Landsat reflectance. Within a three-month window that moved over the FPC stack, we calculated: (i) the median; and (ii) the robust semivariance [57] for pairs of pixels with central locations 30 m apart. These two quantities not only allowed us to minimise the effects of outliers, but they also allowed us to compute a localised coefficient-of-variation for FPC (FPCCV). Note that, to be included in the calculation of FPCCV, all 25 pixels associated with a unique observation date had to be uncontaminated by cloud or cloud shadow [58] or water [59]. By examining FPCCV for large shifts, and also viewing the original Landsat reflectances for context, we identified the year and month of clearing for a site. If the month could not be reliably established, we looked for the narrowest window of months when the clearing likely occurred. In the worst-case scenario, the time of clearing was simply given as entirety of the baseline site’s SLATS reporting period.

Sixty-nine percent of baseline sites were attributed with a single month for the clearing window (Fig. 8a), although 13% were so uncertain that the clearing window could not be narrowed to less than the SLATS reporting period. For an exemplar baseline site—the same site photographed in Fig. 2a—the FPCCV was temporally stable until the land clearing event triggered large fluctuations (Fig. 8b–d). Generally, within our dataset, we found that tree-pulling would induce a sudden decrease in FPC, but also—due to the random alignments of debris, litter, and previously obscured soil—tended to increase the short-range spatial variation of FPC. These two effects are integrated into FPCCV. The slow-acting methods of clearing (Table 2) had only a subtle effect on FPCCV, so the timing of the clearing event was more difficult to detect.

## Abbreviations

CWD: Coarse woody debris
NGGI: National greenhouse gas inventory
FullCAM: Full carbon accounting model
SLATS: Statewide Landcover and Trees Study
GAMM: Generalised additive mixed-effects model
PV: Proportion of variance explained
CCC: Concordance correlation coefficient
PCP: Proportion of validation sites correctly predicted
MAE: Median absolute error
s. c.: Since clearing
FPCCV: Coefficient of variation of foliage projective cover
